# Comparative genomic and biochemical analyses identify a collagen galactosylhydroxylysyl glucosyltransferase from *Acanthamoeba polyphaga mimivirus*

**DOI:** 10.1038/s41598-022-21197-1

**Published:** 2022-10-07

**Authors:** Wenhui Wu, Jeong Seon Kim, Aaron O. Bailey, William K. Russell, Stephen J. Richards, Tiantian Chen, Tingfei Chen, Zhenhang Chen, Bo Liang, Mitsuo Yamauchi, Houfu Guo

**Affiliations:** 1 Department of Molecular and Cellular Biochemistry, University of Kentucky, Lexington, KY USA; 2 Markey Cancer Center, University of Kentucky, Lexington, KY USA; 3 Department of Biochemistry and Molecular Biology, University of Texas Medical Branch, Galveston, TX USA; 4 Department of Biochemistry, Emory University School of Medicine, Atlanta, GA USA; 5 Division of Oral and Craniofacial Health Sciences, Adams School of Dentistry, University of North Carolina at Chapel Hill, Chapel Hill, NC USA; 6 Present Address: Arvinas, LLC, 5 Science Park, New Haven, CT USA

**Keywords:** Biochemistry, Computational biology and bioinformatics, Genetics, Microbiology, Molecular biology

## Abstract

Humans and *Acanthamoeba polyphaga mimivirus* share numerous homologous genes, including collagens and collagen-modifying enzymes. To explore this homology, we performed a genome-wide comparison between human and mimivirus using DELTA-BLAST (Domain Enhanced Lookup Time Accelerated BLAST) and identified 52 new putative mimiviral proteins that are homologous with human proteins. To gain functional insights into mimiviral proteins, their human protein homologs were organized into Gene Ontology (GO) and REACTOME pathways to build a functional network. Collagen and collagen-modifying enzymes form the largest subnetwork with most nodes. Further analysis of this subnetwork identified a putative collagen glycosyltransferase R699. Protein expression test suggested that R699 is highly expressed in *Escherichia coli*, unlike the human collagen-modifying enzymes. Enzymatic activity assay and mass spectrometric analyses showed that R699 catalyzes the glucosylation of galactosylhydroxylysine to glucosylgalactosylhydroxylysine on collagen using uridine diphosphate glucose (UDP-glucose) but no other UDP-sugars as a sugar donor, suggesting R699 is a mimiviral collagen galactosylhydroxylysyl glucosyltransferase (GGT). To facilitate further analysis of human and mimiviral homologous proteins, we presented an interactive and searchable genome-wide comparison website for quickly browsing human and *Acanthamoeba polyphaga mimivirus* homologs, which is available at RRID Resource ID: SCR_022140 or https://guolab.shinyapps.io/app-mimivirus-publication/.

## Introduction

In vertebrates, collagens represent the most abundant protein family forming the extracellular matrix to support and regulate cells and to maintain tissue form and stability^1,2^. At least 28 members of collagen family have been identified and each member likely carries out specific functions^3^. Fibrillar type I collagen is the most abundant member in the family providing most connective tissues with mechanical strength. Type I collagen is a heterotrimeric molecule composed of two a1 and one a2 chains, and it consists of three structural domains: N- and C-terminal non-triple helical domains (N- and C-telopeptides) and the central triple helical domain. As the major collagen component of the basement membrane, type IV collagen is a heterotrimeric network-forming collagen underlying epithelial and endothelial cells and functioning as a barrier between tissue compartments. Type IV collagen contains the N-terminal 7S, a central triple-helical domain, and the globular C-terminal NC1. To perform their functions, collagens acquire a series of specific post-translational modifications (PTMs) during biosynthesis^4^. Collagen prolyl 4-hydroxylation catalyzed by collagen prolyl 4-hydroxylases is critical for stabilizing the triple-helical structure of collagens^5^. Prolyl 3-hydroxylases catalyze prolyl 3-hydroxylation and defects in this minor modification are associated with recessive osteogenesis imperfecta^5^. A series of lysine (Lys) PTMs of collagens are critical for the stability of collagen fibrils. In the cells, Lys residues in the sequences of X-Lys-Gly (helical domain) and X-Lys-Ala/Ser (telopeptides) can be hydroxylated by lysyl hydroxylases 1–3 (LH1-3) to form 5-hydroxylysine (Hyl)^6^. It is generally accepted that LH1 is the main LH for the helical domain and LH2 for the telopeptides. Certain Hyl residues in the collagen helical domain are galactosylated by glycosyltransferase 25 domain containing 1 and 2 (GLT25D1 and GLT25D2) and then glucosylated by lysyl hydroxylases to form a unique Hyl-*O*-linked glycosylation with a mono- or di-saccharide^7–12^. LH3 was believed to be the only galactosylhydroxylysyl glucosyltransferase (GGT) catalyzing collagen glucosylation^8,13^, however, recent studies suggested LH1 and LH2 have GGT activities as well^12,14^. Collagen prolyl and lysyl PTMs are tightly regulated during the development and their alterations lead to various diseases^4,15^. For instance, mutations in the gene encoding LH2 result in Bruck syndrome II a rare osteogenesis imperfecta with joint contracture, but hyper LH2 activities contribute to fibrosis and cancer growth and metastasis^12,16–24^.

Besides multicellular animals, collagen-like proteins and collagen-modifying enzymes are also highly conserved across species and have been found in certain fungi, bacteria, and viruses such as mimivirus^25–28^. Since the initial release of the mimiviral genome^29^, studies have identified 7 mimiviral collagen genes and 2 mimiviral collagen-modifying enzyme genes that encode three enzymes, including collagen prolyl hydroxylase, collagen lysyl hydroxylase, and collagen hydroxylysyl glucosyltransferase^27,30^. Structural and functional studies of mimiviral collagen lysyl hydroxylase provide insights into functions of the human collagen-modifying enzymes^12,31^. Since collagen is widely used for tissue and biomaterial engineering, efforts have been made to generate recombinant collagens using different expression systems^32–34^. Interestingly, a hydroxylated human collagen III fragment has been produced in *Escherichia coli* by coexpressing it with mimiviral collagen prolyl and lysyl hydroxylases^30^. However, glycosylated human collagen is still unable to be produced in the bacterial expression system, at least due in part to the difficulty of expressing active human collagen glycosyltransferases in bacteria.

Mimivirus is the first giant virus discovered and is the prototype and best-characterized virus in the family^29^. The initial mimiviral genome sequencing effort identified 917 protein-encoding genes^29^. These genes play diverse functions in nucleotide and protein biosynthesis, including DNA replication, repair, transcription and translation^35–39^. This effort also identified enzymes involved in various PTMs including 11 glycosyltransferases^29^. As the sequencing technique advances, a later sequencing analysis identified 75 new genes and increased the mimiviral genes to exceed 1000^40^. More recent work identified citric acid cycle and β-oxidation pathway genes in the Mimiviridae family^41,42^. Since the release of the mimiviral genome sequence and the first search for its homology to other species^29^, more than 50 mimiviral proteins have been expressed and characterized (Supplemental Table 1S), which provides valuable insights into virology and raises questions regarding the definition of viruses.

To facilitate the further study of mimiviral homologous proteins, a systematic search of mimiviral homologous proteins in humans was performed. We compared human and mimiviral proteins at the genome-wide level using the DELTA-BLAST (Domain Enhanced Lookup Time Accelerated BLAST)^43^. Besides the initially identified 194 mimiviral ORFs that shared homology with human genes mainly involving in DNA and protein metabolism, we found 52 new mimiviral ORFs that may encode proteins with similarity to these of humans. Eight mimiviral collagen-like proteins (L71, L668, L669, R196, R238, R239, R240, and R241) and 4 putative mimiviral collagen-modifying enzymes (L230, L593, R655, and R699) were identified. To validate the results, we expressed a putative mimiviral collagen glycosyltransferase R699 and showed that R699 glucosylates both free galactosylhydroxylysine and collagen peptidyl galactosylhydroxylysine. These findings suggested that galactosylhydroxylysyl glucosyltransferase is not restricted to the domains of life. Mimiviruses may have the ability to generate Hyl-*O*-linked glycosylation in a similar way as animals. Since mimiviral collagen modifying enzymes are stable in the bacterial expression systems, these enzymes, such as L230 and R699, may be useful to produce recombinant collagen to meet the biomedical research and clinical needs^30^. Moreover, we established an interactive and searchable genome-wide comparison tool (RRID Resource ID: SCR_022140 or https://guolab.shinyapps.io/app-mimivirus-publication/). This user-friendly website helps users quickly browse the protein sequence homology between humans and mimivirus at the genome-wide level for querying new homologs and generating new hypotheses. This website may facilitate the understanding of human-mimivirus interactions during evolution.

## Results and discussion

### Human and mimivirus homology

We used the translation products of 979 mimiviral ORFs to query human homologs in human non-redundant protein sequences using DELTA-BLAST. We set e-value ≤ 0.01, hit span ≥ 35 aa, % identical sequences ≥ 0.25 as cutoffs to include most globular proteins for analysis and minimize the homology bias toward small peptide repeats (such as collagen repeats). Using these cutoffs, 322 queries resulted in at least 1 hit with 41,521 hits in total (Supplemental Table 2S). The search found 4123 unique human RefSeq records in total. Further analysis showed that the 4123 unique human RefSeq records are from 1236 unique human proteins (Supplemental Table 2S). To increase the robustness of the search, we used 4123 unique human RefSeq records that we identified in the first round of analysis to search for mimiviral homologs. Using the same filtering standard, we found that 3325 RefSeq queries or 1049 human proteins result in at least 1 hit and 58,011 hits in total (Supplemental Table 3S). This search found 307 mimiviral protein sequences, 265 of which overlap with the 322 mimiviral queries that we started with. Of these 265 mimiviral queries, 52 of them are newly identified (Supplemental Table 4S). Mimiviral L393 shares the highest level of amino acid sequence identity (> 62%) with human heat shock 70 kDa protein 1-like. The most common motif shared by humans and mimivirus is ankyrin repeats. Besides the 48 mimiviral ankyrin repeats previously identified, our analyses added 33 more mimiviral proteins with ankyrin repeats.

**Figure 1:**
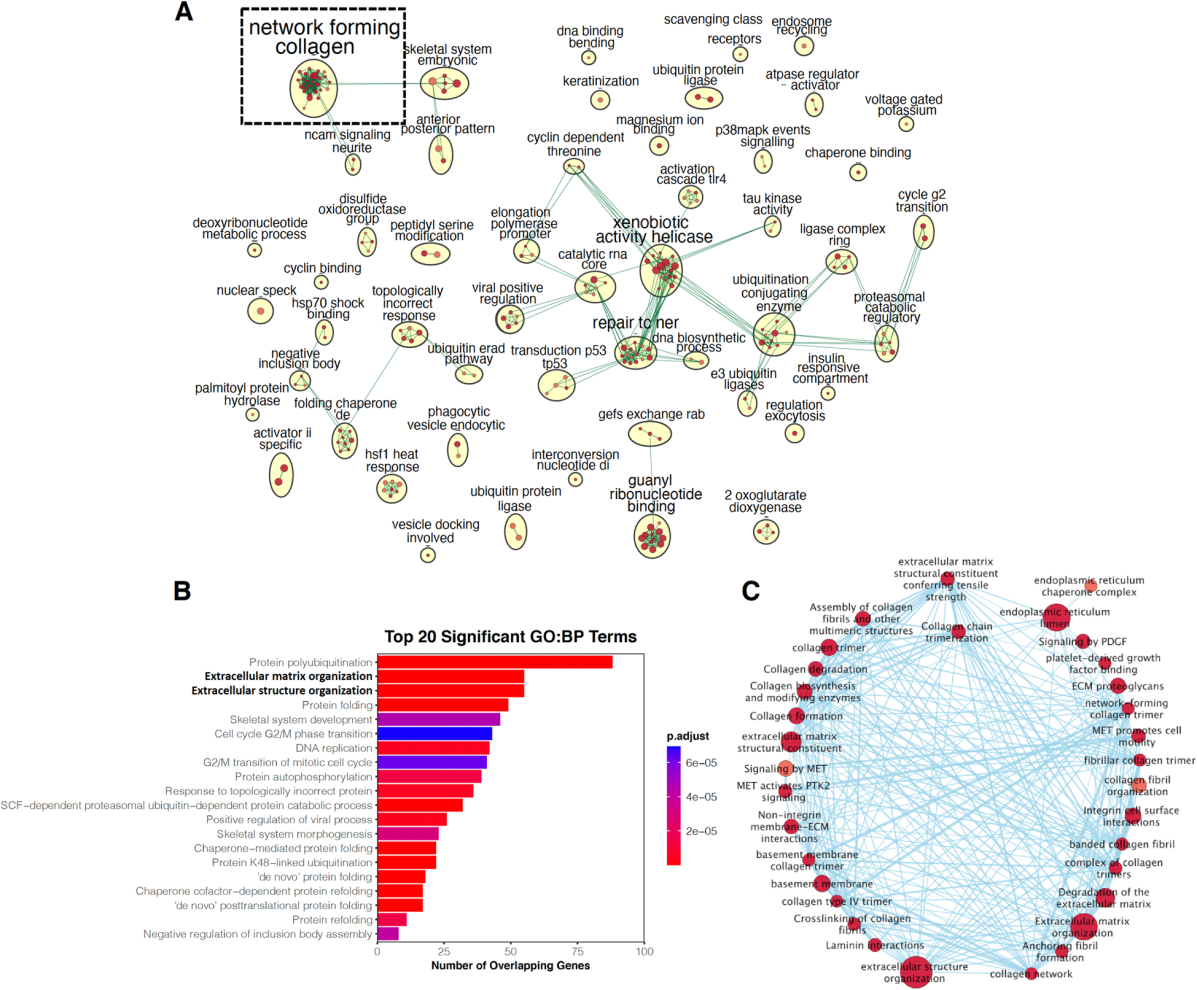
Comparative genomic analysis of humans and mimivirus. (**A**) Functional enriched Gene Ontology (GO) and REACTOME pathways that are shared between humans and mimivirus. We performed a genome-wide search of homologous proteins in human genome using the translation products of mimiviral ORFs as queries. This search identified 4123 unique human RefSeq records that were organized into GO and REACTOME pathways. Gene ontology networks were built based on the results from pathway analysis and visualized using *Cytoscape3.8.2.* Collagen related networks were highlighted with a dashed black square. (**B**) Collagen related pathways form the largest subnetwork with the most nodes. Top 20 GO enriched biological processes were shown. Collagen-related pathways are the major components of Extracellular matrix organization and Extracellular structure organization. Collagen related pathways were highlighted in bold. (**C**) Subnetwork analysis shows the organization of collagen-related pathways.

To identify the enriched pathways conserved between humans and mimivirus, we performed GO and REACTOME pathway analyses using an adjusted p-value ≤ 0.05 as a cutoff (Supplemental Tables 5S–8S). GO pathways were organized based on cellular component, molecular function, and biological process (Supplemental Tables 5S–7S). Functional enriched GO and REACTOME pathways were then used to build gene ontology networks and visualized using *Cytoscape3.8.2* (Fig. 1A). This analysis identifies 52 clusters involved in endocytosis, ubiquitination, DNA and collagen metabolism. The largest cluster with the most nodes is the network forming collagen composed of collagen and collagen-modifying enzymes (Fig. 1A). Collagen-related pathways rank high in both GO and REACTOME pathway analyses (Fig. 1B and Supplemental Tables 5S–8S).

### Homology in collagen and collagen-modifying enzymes

**Figure 2:**
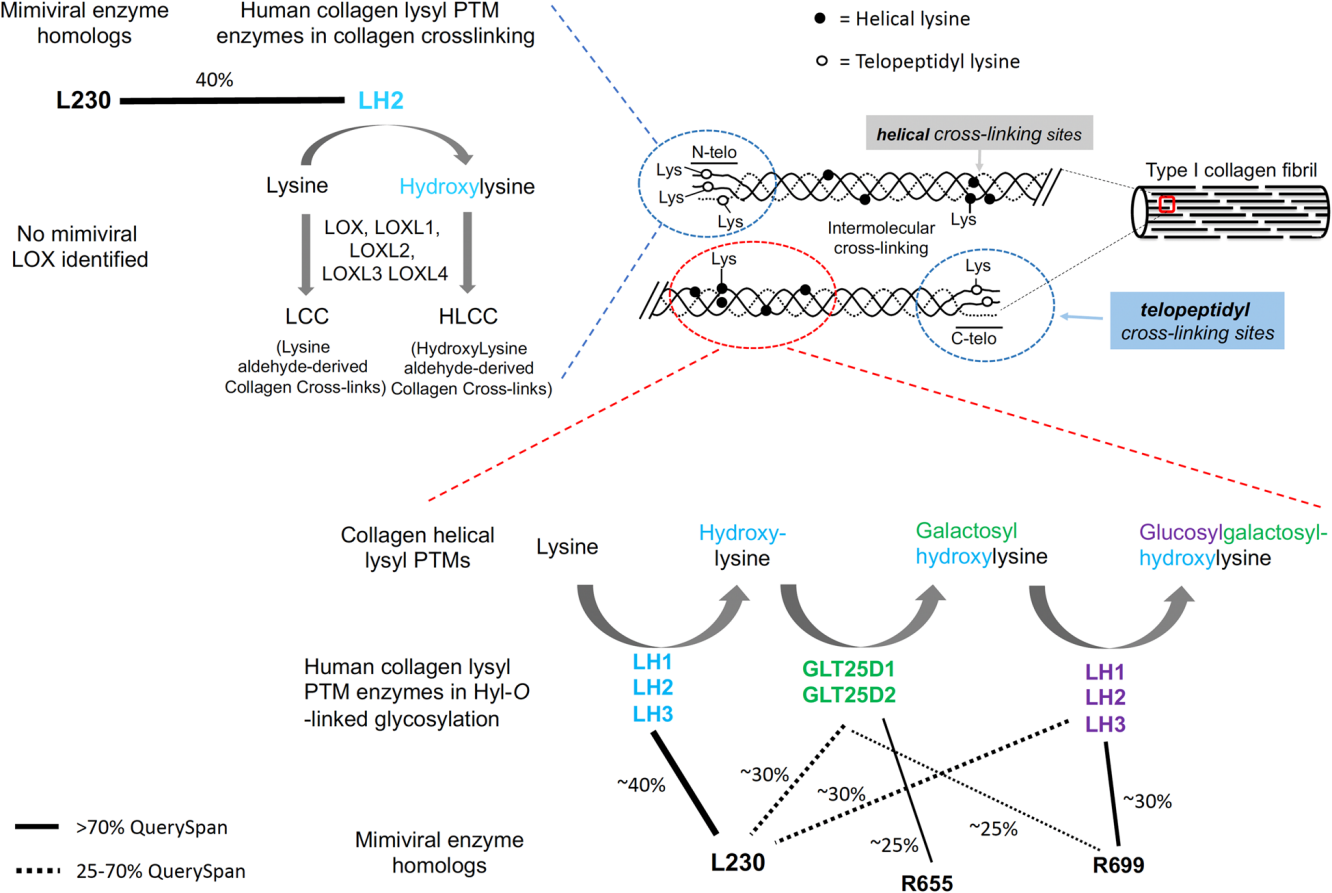
Schematics showing collagen lysyl PTMs pathway and its homology to mimiviruses. Sequence alignments suggested that mimiviral L230, R699 and R655 are homologous to human collagen lysyl modifying enzymes involved in collagen crosslinking and Hyl-*O*-linked glycosylation. A newly identified mimiviral putative collagen glycosyltransferase R699 shares higher amino acid sequence identity (~ 30%) with human collagen GGTs than hydroxylysyl galactosyltransferases (GLT25D1 and GLT25D2). Homology was indicated with black lines (solid black lines if percentage of QuerySpan was higher than 70%, and dashed black lines if percentage of QuerySpan fell between 25% and 70%) and the percentage of amino acid sequence identity was shown.

We defined minimal length, hit span, etc. during comparative genomic analyses to minimize the homology bias toward small peptide repeats (such as collagen repeats). Interestingly, collagen related pathways still formed the largest networks. We thus focused on network forming collagen for further analysis (Fig. 1C). Further analysis showed that the human protein hits we identified using mimiviral queries are involved in collagen biosynthesis and assembly. Our search identified 8 mimiviral collagens (L71, L668, L669, R196, R238, R239, R240, and R241) and 4 mimiviral collagen-modifying enzymes (L230, L593, R655, and R699, Fig. 2). Of these 12 collagen and collagen-modifying enzyme genes, the identities of R238, R655, and R699 have not been revealed. Sequence homology analyses suggested that R238 is a collagen-like protein, while R655 and R699 are putative collagen glycosyltransferases (Fig. 2) with moderate homology to human GLT25D and LH family members, respectively. Eight of the mimiviral collagen-related proteins identified by the initial search were not correctly annotated by previous work^29^. For instance, 4 mimiviral collagen-like proteins (R196, L669, R239, and R241) had been annotated as PPE-repeat proteins and 2 putative collagen-modifying enzymes (L230 and R655) as LPS biosynthesis enzymes^29^. Of these mis-annotated mimiviral proteins, L230 was expressed and characterized as collagen telopeptidyl lysyl hydroxylase and collagen hydroxylysyl glucosyltransferase (Fig. 2)^27,31^. Structural and mutagenesis analyses suggested that L230 lysyl hydroxylase domain forms a Fe^2+^-stabilized tail-to-tail homodimer^31^, similar to human LH family members. For the mimiviral proteins that were not annotated during the initial release of the mimiviral genome, L71 was confirmed to be a type of mimiviral collagen that may play a role in the pathogenesis of arthritis in humans^44^. L593 was shown to hydroxylate human type III collagen proline residue and was used to generate a human recombinant collagen III fragment in a bacterial expression system^30^. These results validate our search of the viral collagen and collagen-modifying enzymes, suggesting that our analyses are robust and relevant. No lysyl oxidase or transglutaminase was identified, suggesting that mimiviral collagens are either crosslinked by host enzymes or not crosslinked at all.

### R699 has collagen GGT activity

**Figure 3:**
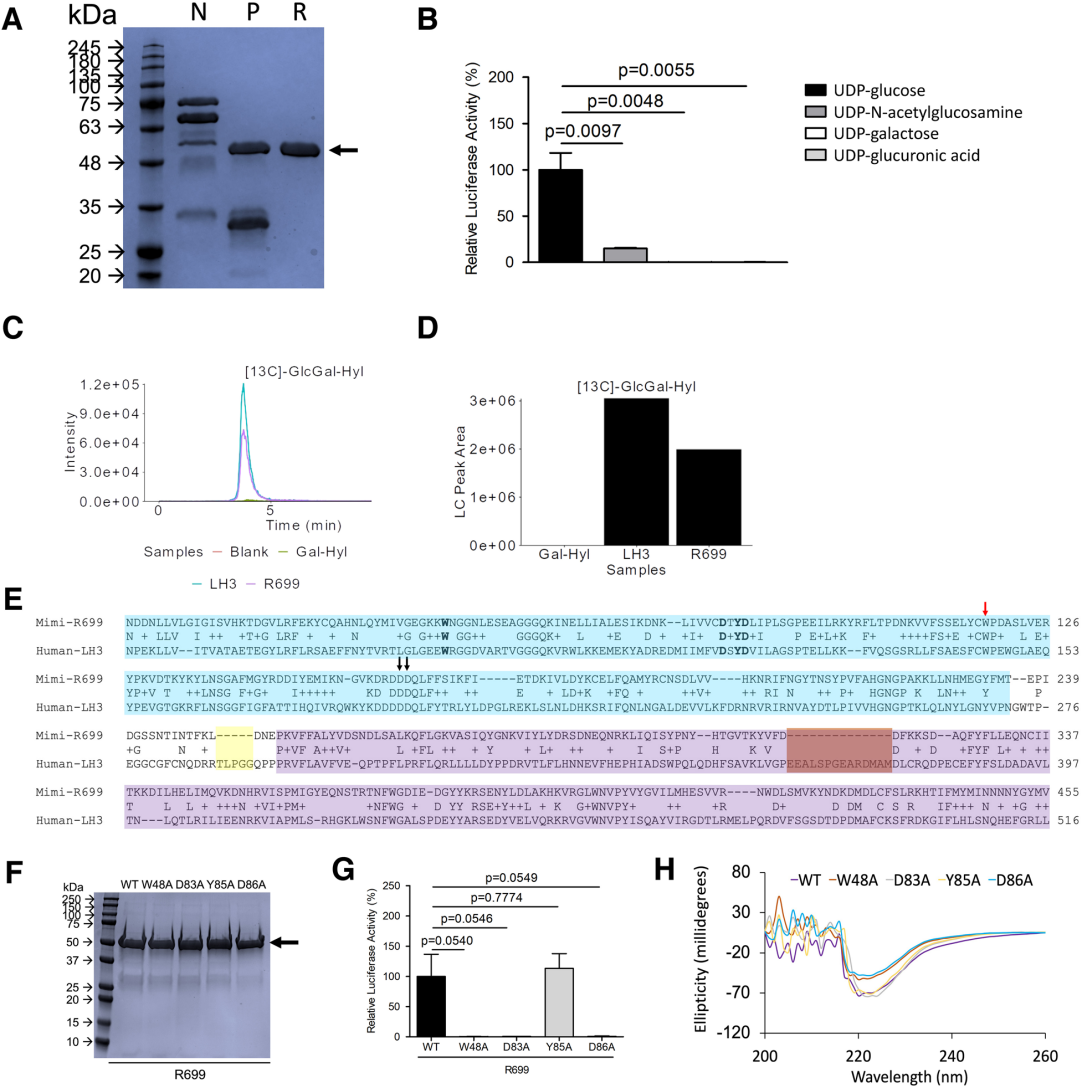
R699 is a GGT. (**A**) SDS–polyacrylamide gel electrophoresis of R699 protein after immobilized metal affinity chromatography (IMAC) with nickel resin (N), HRV 3C protease cleavage (P), reverse IMAC (R). R699 was purified close to homogeneity after 3-step purification. The approximate size of the recombinant protein was indicated with an arrow. Gel image was recolored. (**B**) R699 GGT activity was assayed using an adenosine triphosphate-based luciferase assay. Substrate was galactosylhydroxylysine. GGT activity was measured by detecting UDP production. Mean ± SD of 3 replicates, *p* values, two-tailed Student’s *t* test. (**C**) and (**D**) R699 GGT assay was performed using UDP-[UL-^13^C_6_] glucose. Carbon-13 labeled glucosylgalactosyl-hydroxylysine ([^13^C]GlcGal-Hyl) was confirmed with LC–MS analysis. The amount of [^13^C]GlcGal-Hyl was determined based on LC peaks in Fig. C using MultiQuant software (SCIEX). Chromatograms (**C**) and bar graph (**D**) were plotted using custom R scripts. Galactosyl-hydroxylysine was shown as Gal-Hyl. n = 1. (**E**) Sequence alignment of R699 with human LH3. Residues within the GGT and accessory (AC) domains were labeled in cyan and purple, respectively. Asp190 and Asp191 in poly-Asp repeat and Trp145 were indicated with arrows (residue number based on LH3 sequence). The interdomain loop deletion and the largest deletion in R699 were highlighted in yellow and brown squares, respectively. (**F**) SDS–polyacrylamide gel electrophoresis of R699 wild type (WT) and mutant recombinant proteins. The approximate size of the recombinant proteins was indicated with an arrow. Gel image was recolored. (**G**) GGT activity of WT and mutant R699 recombinant proteins was assayed using Gal-Hyl as substrate. The readout of the assay is adenosine triphosphate production which was detected using an adenosine triphosphate-based luciferase assay. Mean ± SD of 3 replicates, *p* values, two-tailed Student’s *t* test. (**H**) Circular dichroism spectrometry of wild-type R699 (WT) and R699 mutants.

Of the two new putative collagen-modifying enzymes, R655 was speculated to be a putative mimiviral glycosyltransferase^27^ while little is known about R699. As a result, we selected R699 for further biochemical analyses. We hypothesized that R699 is a collagen GGT because R699 shows a higher sequence similarity to human collagen GGTs than collagen hydroxylysyl galactosyltransferases (Fig. 2, 29.3% amino acid sequence identity with human LH2, E value = 6e−60). To generate R699 recombinant protein for biochemical analyses, we synthesized the R699 gene with an HRV 3C protease cleavable N-terminal His_6_ and mCherry tags and expressed it in *Escherichia coli*. We found that R699 produces a stable soluble protein (Fig. 3A and Supplemental Fig. 1S) after overnight Isopropyl β-D-1-thiogalactopyranoside-indued expression at 16 °C. We lysed the *Escherichia coli* cells via sonication and purified the R699 protein with immobilized metal affinity chromatography (IMAC) using nickel resin. N-terminal His_6_ and mCherry tags were cleaved by HRV 3C protease and removed by reversed IMAC. Highly purified R699 protein was obtained after the 3-step purification procedure (Fig. 3A and Supplemental Fig. 1S) with a yield of ~ 10 mg per liter of *E. coli* culture.

Given the moderate sequence similarity between R699 and human GGTs, we hypothesized that R699 functions as a collagen GGT. To test this possibility, we reacted R699 with amino acid substrate galactosylhydroxylysine using UDP-glucose and 3 other sugar donors. Glycosylation was measured by detecting UDP production with a luciferase-based assay as we previously described^12^. Under these conditions, R699 showed robust activity with UDP-glucose but no other sugar donors (Fig. 3B). We also performed the GGT enzymatic activity assay using UDP-[UL-^13^C_6_]-glucose as a sugar donor to confirm the glucosylation events by liquid chromatography-mass spectrometry (LC–MS) analysis. The glucosylation of galactosylhydroxylysine to [^13^C_6_]-glucosylgalactosylhydroxylysine was detected in the presence of R699 (Fig. 3C,D) or a known collagen GGT (recombinant human LH3). [^13^C_6_]-glucosylgalactosylhydroxylysine was not detected in buffer or galactosyl-hydroxylysine (Gal-Hyl) negative control.

By comparing with the LH3 catalytic domain, we found that the R699 Mn^2+^-binding DXXD motif and UDP-binding Trp and Tyr residues are strictly conserved (Fig. 3E). Site-directed mutagenesis and enzymatic activity assay showed that DXXD is critical for R699’s GGT activity (Fig. 3F,G, and Supplemental Fig. 2S). Interestingly, Trp but not Tyr is critical for GGT activity (Fig. 3F,G), suggesting Trp is the primary residue engaging UDP. Circular dichroism spectra suggested D83A and Y85A show similar spectra as the wild type (Fig. 3H) while W48A and D86A are slightly different, suggesting D83A and Y85A are not deleterious to R699 secondary structure.

Other residues critical for collagen GGT function are conserved in R699 as well. Asp190 and Asp191 in the poly-Asp repeat of LH3 that was suggested to be involved in catalysis^45^ are conserved (Fig. 3E, black arrows). A unique Trp145 in LH3 that was thought to be a gating residue is also conserved (Fig. 3E, red arrow). Sequence alignment suggested that the R699 inter-domain loop has a 5-residue deletion (Fig. 3E highlighted in yellow) and lacks the cysteine-linked hairpin structure (Fig. 3E). The largest 14-residue deletion (Fig. 3E highlighted in brown) occurs in the accessory domain that is not required for LH3’s GGT activity but modulates LH2’s GGT activity^12,46^. These findings suggest the key residues involved in collagen GGT activity are conserved in R699.

**Figure 4:**
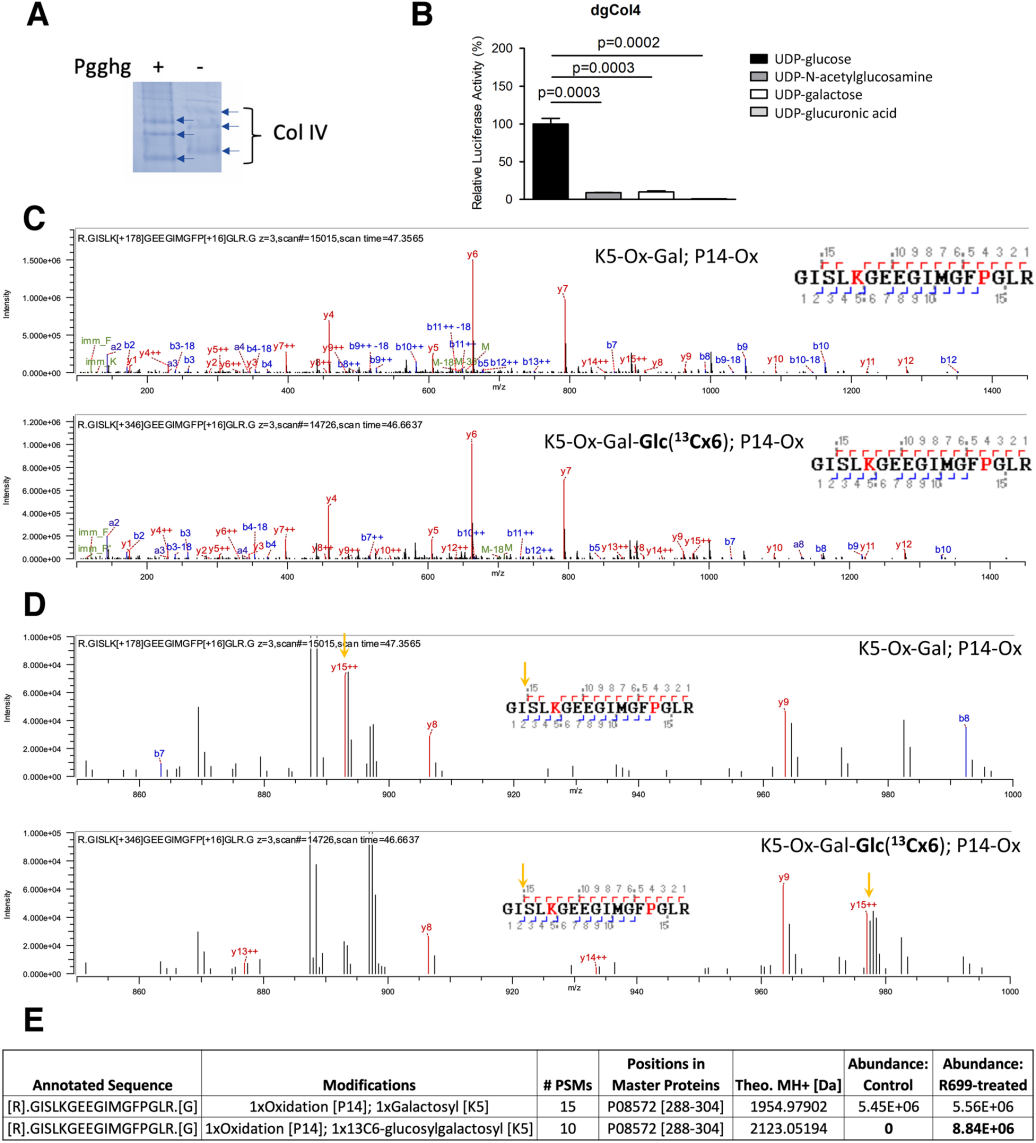
R699 is a collagen GGT. (**A**) Type IV collagen that had been pre-treated with wild-type (+) protein-glucosylgalactosylhydroxylysine glucosidase (PGGHG) or sham-treated (-) was analyzed using SDS–polyacrylamide gel electrophoresis. Gel image was recolored. (**B**) R699 collagen GGT activity was assayed. Substrate was deglucosylated type IV collagen from A and GGT activity was measured similarly as in Fig. 3B. Mean ± SD of 3 replicates, *p* values, two-tailed Student’s *t* test. (**C**) MS/MS analysis of type IV collagen. R699 GGT assay was performed using deglucosylated type IV collagen and UDP-[UL-^13^C_6_] glucose. Peptides containing galactosyl-hydroxylysine (Gal-Hyl, top) or Carbon-13 labeled glucosyl-galactosylhydroxylysine ([^13^C]GlcGal-Hyl, bottom) were detected by MS/MS. The spectra exhibit peaks corresponding to b and y ion series from fragmentation of each peptide. Peptide sequences with the identified fragment ions were indicated in the upper right. The b and y ions were labeled in the spectra and indicated on the peptide sequence in upper right. (**D**) Zoom-in of spectra in (**C**) showing y15++. Peptide sequences, b and y ions were labeled similarly as in (**C**). The locations of y15++ in the spectra and peptide sequences were highlighted with yellow arrows. (**E**) The details about peptide sequences, modifications, and abundance were summarized.

To test whether R699 is a collagen peptidyl GGT, we reacted R699 with deglucosylated type IV collagen substrate (Fig. 4A,B) using UDP-glucose and 3 other sugar donors. Deglucosylation of type IV collagen was generated as we previously described (Fig. 4A and Supplemental Fig. 3S) ^12^. Under these conditions, R699 showed robust activity in the presence of UDP-glucose but no other sugar donors (Fig. 4B). We also performed the GGT enzymatic activity assay using UDP-[UL-^13^C_6_]-glucose as a sugar donor to confirm the glucosylation events on type IV collagen by LC–MS (Fig. 4C–E). LC–MS analyses suggested that R699 (Fig. 4C,D) glucosylates collagen peptidyl galactosylhydroxylysine to [^13^C_6_]-glucosylgalactosylhydroxylysine. The MS/MS spectra exhibited peaks corresponding to b and y ion series from fragmentation of peptides (aa288-304 of Col4a2) containing galactosylhydroxylysine (top spectrum) or [^13^C_6_]-glucosylgalactosylhydroxylysine (bottom spectrum). The b and y ion series were unambiguously identified and assigned. Enlarged spectra of the peptides in Fig. 4C showed the y15++ shift upon glucosylation (Fig. 4D, y15++ highlighted with arrows). The relative abundance quantification results suggested ^13^C_6_]-glucosylgalactosylhydroxylysine is only present in the R699 treated deglucosylated type IV collagen sample but undetectable in untreated control (Fig. 4E), which is consistent with the luciferase-based enzymatic activity assay results (Fig. 4B). These findings suggested R699 is a mimiviral collagen GGT.

It was reported that mimiviral L230 functions as a collagen LH and a hydroxylysyl glucosyltransferase to produce peptidyl glucosylhydroxylysine^27^, thus it is tempting to test the possibility of R699 modifying peptidyl glucosylhydroxylysine. Toward this end, recombinant L71 containing Hyl was produced by co-expressing L71 and L230 in *Escherichia coli*. L71 was then isolated and glucosylated by purified recombinant L230 using UDP-glucose as the sugar donor. L71 containing glucosylhydroxylysine was extensively dialyzed before reacting with R699. R699-catalyzed glucosylation reaction was detected with a luciferase-based assay. However, no luciferase activity was found (data not shown). These findings do not support that R699 functions as a peptidyl glucosylhydroxylysine glucosyltransferase. Our work suggests that R699 acts on peptidyl galactosylhydroxylysine. The source of peptidyl galactosylhydroxylysine remains to be determined. It may be generated by the host or by an unknown mimiviral collagen hydroxylysyl galactosyltransferase. Since R655 shares moderate amino acid sequence identity (23%) with a human collagen hydroxylysyl galactosyltransferase GLT25D1, it warrants analysis of R655’s collagen hydroxylysyl galactosyltransferase activity.

### Website features and functionalities

**Figure 5:**
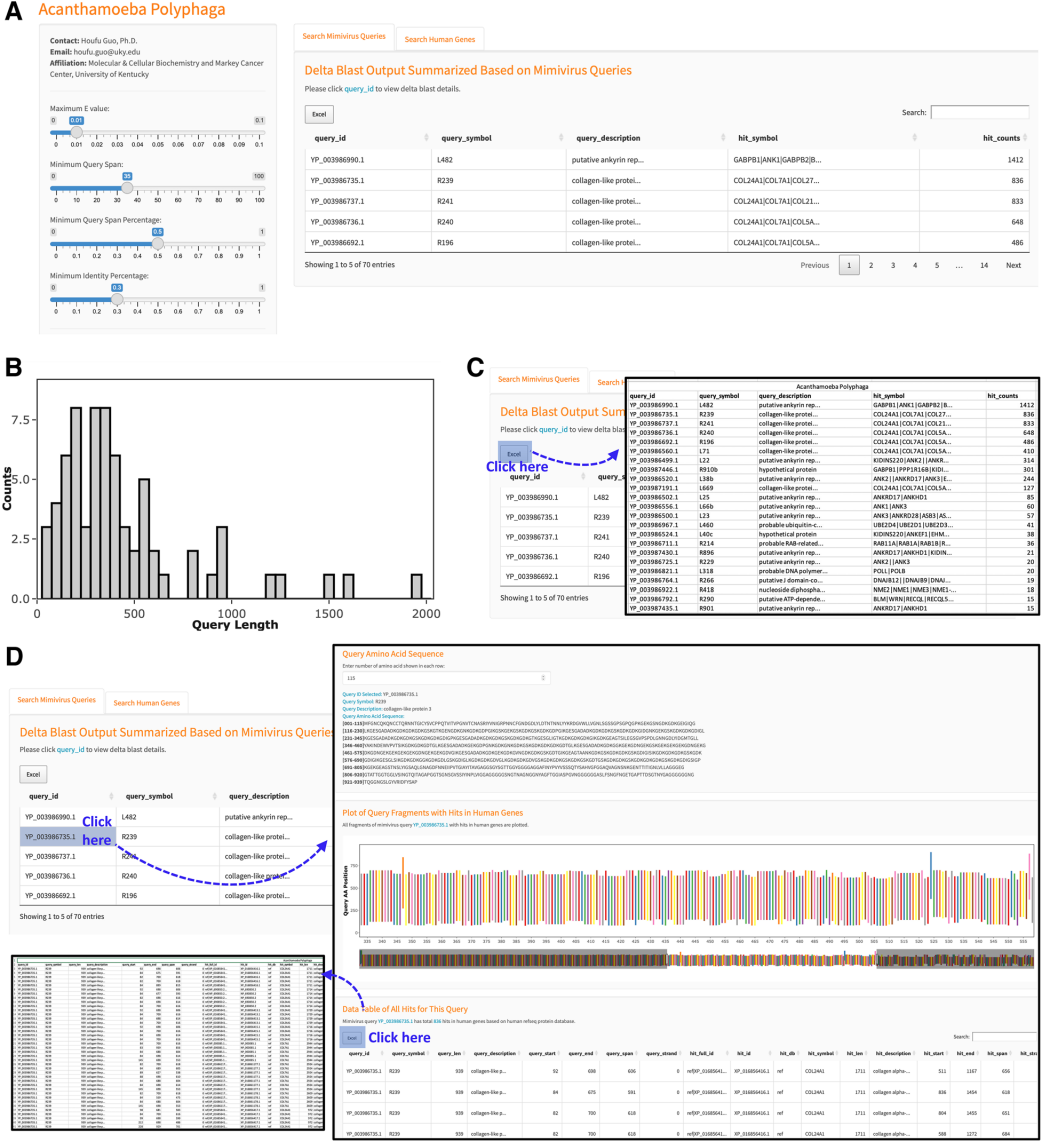
Human and mimivirus homology tool features and functionalities. (**A**) An interactive tool was established for easily searching and browsing of human and mimiviral homolog proteins. (**B**) Bar graph showing the overall distribution of homologous proteins. (**C**) After performing search, clicking Excel (highlighted with a blue square) to download a list of human or mimivirus homologous proteins. (**D**) Clicking on the query_id to show the details of the query and search (the Gene ID, symbol, description and sequence). Under the section of Data Table of All Hits, an excel file with the details of the search is available for download.

To facilitate the further analysis of the homology between humans and mimivirus, we established an interactive tool for easily searching and browsing of human and mimiviral homolog proteins (RRID Resource ID: SCR_022140 or https://guolab.shinyapps.io/app-mimivirus-publication/). Users can modify the search by changing the E value (Maximum Evalue), the length of query span in amino acid (Minimum Query Span) or percentage (Minimum QuerySpan Percent), the sequence identity percentage (Minimum Identity percentage) (Fig. 5A). The overall distribution of homologous proteins is shown in a histogram as counts vs. query length (Fig. 5B). If a list of all homologous proteins is needed, modify the search criteria without inputting a query. Clicking Excel on “Search Mimivirus Queries” tab or “Search Human Queries” tab will download an excel file including a list of all mimivirus or human homologous proteins, respectively (Fig. 5C). Search can be performed by inputting a query ID, gene name, or keywords about the query description. By clicking on the query_id, the details of the search (the Gene ID, symbol, description, and sequence) will be shown (Fig. 5D). The list of homologous hits is shown as a table under the Data table. The search details can be downloaded as an Excel file by clicking Excel (Fig. 5D).

## Conclusions

In conclusion, we performed genome-wide DELTA-BLAST of human and mimivirus genomes and found 52 new mimiviral ORFs that may encode proteins with similarity to these of humans. To identify the potential functions of mimiviral ORFs, we performed Gene Ontology (GO) and REACTOME pathway analyses. The analyses showed that collagen and collagen-modifying enzymes form the largest subnetwork with most nodes. Two new putative glycosyltransferases, R655 and R699, were found in the mimiviral collagen-related pathways. Protein biochemical analyses confirmed that R699 is a new mimiviral collagen galactosylhydroxylysyl glucosyltransferase, suggesting our search is robust. We established an interactive and searchable genome-wide comparison tool (RRID Resource ID: SCR_022140 or https://guolab.shinyapps.io/app-mimivirus-publication/). This tool is established based on the DELTA-BLAST results that helped us identify more homologous proteins. The interactive and searchable nature of the website allows the users to modify the search criteria and quickly browse human and mimivirus homologous proteins with different levels of homology at the genome-wide level.

## Methods

### Comparative genome-wide analysis of human and mimivirus homologous proteins

To search mimiviral protein sequences against the human non-redundant protein sequence database, we installed and ran the DELTA-BLAST command line application in Lipscomb Compute Cluster at the University of Kentucky with default parameters. We obtained Acanthamoeba Polyphaga Mimivirus GCF_000888735.1 assembly and annotation data from NCBI’s RefSeq ftp site ftp://ftp.ncbi.nlm.nih.gov/genomes/all/GCF/000/888/735/GCF_000888735.1_ViralProj60053. The file “*GCF_000888735.1_ViralProj60053_protein.faa.gz”* that contains 979 protein sequences was used as the input. All mimiviral protein sequences were searched against the human non-redundant protein sequence database, which was downloaded from NCBI’s blast ftp site https://ftp.ncbi.nlm.nih.gov/blast/db/. The file “GCF_000888735.1_ViralProj60053_feature_table.txt.gz” containing the feature information for all mimiviral protein sequences was used for annotation.

The DELTA-BLAST output in *xml* format was parsed and all high-scoring pairs (written as hits) were constructed into a tubular format using the biopython package *Bio. SearchIO.* The resulted data table was further processed in *R*. Among 979 mimiviral protein queries, 808 queries had at least one hit and 556,603 hits were found in total. Using e value ≤ 0.01, hit span ≥ 35 amino acids, and the percentage of identical sequences between query and hit ≥ 0.25 as cutoffs, 356 query sequences with 85,881 total hits passed the defined criteria. These 356 query sequences share sequence similarity with 4123 unique human RefSeq records in total.

To confirm the similarity mapping between mimivirus and human protein sequences, 4123 unique human RefSeq protein records we identified were DELTA-BLAST searched against the mimivirus non-redundant protein sequence database. The human RefSeq protein sequence file “GCF_000001405.39_GRCh38.p13_protein.faa.gz” of the latest GRCh38 assembly was downloaded from NCBI’s RefSeq ftp site ftp://ftp.ncbi.nlm.nih.gov/genomes/refseq/vertebrate_mammalian/Homo_sapiens/latest_assembly_versions/GCF_000001405.39_GRCh38.p13. Four RefSeq records were not present in the file therefore manually checked for their sequences in NCBI. A newly formed file containing all 4123 protein sequences of interest was used for DELTA-BLAST command line application with default parameters. After DELTA-BLAST, the output was processed in the same manner as the first DELTA-BLAST search. Using the same cutoffs, we found that 3325 human RefSeq records (1049 unique gene symbols) have hits in 307 unique mimiviral protein sequences. Of these 307 unique mimiviral protein sequences, 265 of them overlap with the 322 mimiviral queries that we identified in the first round of search. To summarize the results for overlapping mimiviral sequences, 1031 human genes and their corresponding mimiviral sequences were organized and presented (Supplementary Table 9S).

### Enrichment map and network

To reduce the redundant hits between different databases, we selected the hits from the RefSeq database to perform pathway enrichment analysis using HUGO gene symbol, which resulted in 322 queries with at least one hit and 41,520 hits in total. At the protein level, these 41,520 hits are from 4123 unique RefSeq records, which contains 2027 proteins (IDs prefix with NP) and 2096 predicted proteins (IDs prefix with XP). These RefSeq record IDs were then converted into HUGO gene symbols using Bioconductor package *biomaRt*. The ones that could not be converted by *biomaRt* were manually checked for the corresponding gene symbols in Genecard and BioGPS. Eventually, this conversion resulted in 1236 unique gene symbols, which were then used for pathway enrichment analysis and building Shiny App for visualization. The Shiny App is hosted on shinyapps.io server and is publicly available (https://guolab.shinyapps.io/app-mimivirus-publication/). This resource was submitted to RRID Portal with a Resource ID: SCR_022140.

To understand the overall biological and biochemical processes that the hits may be involved in, pathway enrichment analysis was performed using the R package *gprofiler2.* The significant GO and REACTOME pathways (adjusted p-value ≤ 0.05) with term sizes between 5 and 350 were selected for constructing pathway networks using *EnrichmentMap*^47^. The resulted clusters were then automatically defined and summarized into major biological themes using *AutoAnnotate*^48^*.* Finally, collagen-related pathways which formed the largest subnetwork were presented separately. All three steps were performed in *Cytoscape3.8.2*^49^*.*

### Cloning, expression, and purification of R699 and variants

R699 gene was synthesized (Genscript). For enzymatic activity assay, R699 was cloned into a modified version of the pET28 vector using BamH1 and EcoR1 sites. This modified version of pET28 has HRV 3C protease and BamH1 recognition sites inserted to replace the thrombin recognition site. The endogenous BamH1 site was destroyed. Mutant constructs were generated using QuickChange Lightning Site-Directed Mutagenesis Kit (Agilent). For SDS-PAGE in Fig. 3A, R699 was also cloned into a version of pET28-mCherry vector using BamH1 and EcoR1 sites. This pET28-mCherry vector has mCherry gene sequence and HRV 3C protease recognition site inserted between Nhe1 and BamH1 sites. All plasmids were verified by sanger sequencing and transformed into *E. coli* BL21 (NEB) for protein expression. Small scale R699-BL21 overnight culture with 50 mg per liter of kanamycin (GoldBio) was prepared and 10 ml of small-scale overnight culture was used to inoculate 800 ml large scale culture using Terrific Broth Medium (Alpha Biosciences) in the presence of the same amount of kanamycin. Culture was grown at 37 °C to OD_600_ = 1.5, chilled on ice for 15 min, induced with 1 mM isopropyl β-d-1-thiogalactopyranoside (IPTG, GoldBio) and grown at 16 °C for 18 h. Cells were collected, pelleted and then resuspended in binding buffer (20 mM Tris, pH 8.0, 200 mM NaCl and 15 mM imidazole). The cells were lysed by sonication and then centrifuged at 23,000 g for 15 min. The recombinant R699 proteins (wild type or mutants) were purified with immobilized metal affinity chromatography and eluted with elution buffer (200 mM NaCl and 300 mM imidazole, pH 8.0) unless stated otherwise. For enzymatic activity assay, R699 protein was dialyzed at 16 °C for 18 h in 20 mM HEPES, pH 7.4, 150 mM NaCl.

### GGT enzymatic activity assay

GGT activity was measured similarly as previously described^12^. The assay was performed in reaction buffer (100 mM HEPES buffer pH 8.0, 150 mM NaCl) at 37 °C for 1 h with 1 μM R699 enzyme, 100 μM MnCl_2_, 200 μM UDP-glucose (MilliporeSigma, St. Louis, MO), 1 mM dithiothreitol and 1.75 mM galactosyl hydroxylysine (Gal-Hyl, Cayman Chemical, Ann Arbor, MI) or 2 μM deglucosylated collagen IV. Deglucosylated collagen IV was generated using a glycosidase PGGHG as previously described^12^. GGT activity was measured by detecting UDP production with an ATP–based luciferase assay (UDP-Glo™ Glycosyltransferase Assay, Promega, Madison, WI) according to manufacturers' instructions. Experiments were performed in triplicate from distinct samples, and an unpaired t-test was used to compare the enzymatic activity of different samples. The glucosylation of galactosyl hydroxylysine was further confirmed by mass spectrometry.

### Mass spectrometry

To confirm the glucosylation of Gal-Hyl and type IV collagen by R699, the R699 GGT assay was performed similarly as discussed above, except that UDP-glucose was replaced with the same concentration of UDP-[UL-^13^C_6_] glucose (Omicron Biochemicals, Inc). LC–MS analysis was used to detect [^13^C]glucosyl-galactosylhydroxylysine. LH3 catalyzed GGT activity assay was used as a positive control. For LC–MS analysis of [^13^C]glucosyl-galactosylhydroxylysine, R699 and LH3 assay samples were diluted to ~ 1 μM in 50% acetonitrile containing 0.1% formic acid. LC–MS analysis was performed using a 1260 Infinity UHPLC System (Agilent) coupled to a Qtrap 6500 mass spectrometer (SCIEX). Samples were separated on a Kinetex EVO C18 column (Phenomenex) with mobile phases included: A) water + 0.1% formic acid, B) acetonitrile + 0.1% formic acid. LC peaks were integrated using MultiQuant 3.0.3 software (SCIEX, https://sciex.com/products/software/multiquant-software). Peak areas and chromatograms were plotted using custom R scripts. Experiments were performed once.

To confirm peptidyl GGT activity, 25 μg of type IV collagen and collagen GGTs from each GGT reaction mixture was solubilized with 5% SDS, 50 mM TEAB, pH 7.55, final volume 25 μl. The sample was then centrifuged at 17,000 g for 10 min to remove any debris. Proteins were reduced by making the solution 20 mM TCEP (Thermo, #77,720) and incubated at 65 °C for 30 min. The sample was cooled to room temperature and 1 μl of 0.5 M iodoacetamide acid added and allowed to react for 20 min in the dark. 2.75 μl of 12% phosphoric acid was added to the protein solution. 165 μl of binding buffer (90% Methanol, 100 mM TEAB final; pH 7.1) was then added to the solution. The resulting solution was added to S-Trap spin column (protifi.com) and passed through the column using a bench top centrifuge (30 s spin at 4000×*g*). The spin column was washed with 400 μl of binding buffer and centrifuged. This was repeated two more times. Trypsin was added to the protein mixture in a ratio of 1:25 in 50 mM TEAB, pH 8, and incubated at 37 °C for 4 h. Peptides were eluted with 80 μl of 50 mM TEAB, followed by 80 μl of 0.2% formic acid, and finally 80 μl of 50% acetonitrile, 0.2% formic acid. The combined peptide solution was then dried in a speed vac and resuspended in 2% acetonitrile, 0.1% formic acid, 97.9% water and placed in an autosampler vial.

Peptide mixtures were analyzed by nanoflow liquid chromatography-tandem mass spectrometry (nanoLC-MS/MS) using a nano-LC chromatography system (UltiMate 3000 RSLCnano, Dionex), coupled on-line to a Thermo Orbitrap Fusion mass spectrometer (Thermo Fisher Scientific, San Jose, CA) through a nanospray ion source (Thermo Scientific). A trap and elute method was used. The trap column was a C18 PepMap100 (300 μm × 5 mm, 5 μm particle size) from ThermoScientific. The analytical column was an Acclaim PepMap 100 (75 μm × 25 cm) from (Thermo Scientific). Peptides were eluted using a 120 min gradient (mobile phase A = 0.1% formic acid (Thermo Fisher), mobile phase B = 99.9% acetonitrile with 0.1% formic acid (Thermo Fisher); hold 12% B for 5 min, 2–6% B in 0.1 min, 6–25% in 100 min, 25–50% in 15 min) at a flow rate of 350 nl min^−1^. Eluted peptide ions were analyzed using a data-dependent acquisition (DDA) method with resolution settings of 120,000 and 15,000 (at *m/z* 200) for MS1 and MS2 scans, respectively. DDA-selected peptides were fragmented using stepped high energy collisional dissociation (27, 32, 37%).

Tandem mass spectra were extracted and charge state deconvoluted by Proteome Discoverer (Thermo Fisher, version 1.4.1.14). Deisotoping was not performed. All MS/MS spectra were searched against a Uniprot Ecoli and human databases as background, and a custom database made of common contaminants and collagen proteins using Sequest and MS Amanda search engines. Searches were performed with a parent ion tolerance of 10 ppm and a fragment ion tolerance of 0.02 Da. Trypsin was specified as the enzyme, allowing for two missed cleavages. Fixed modification of carbamidomethyl (C) and variable modifications of oxidation (M, K, and P), ^13^C_6_-glucosylgalactosyl (+ 346.121 Da (K)), Galactosyl + 178.048 Da (K), and Glucosylgalactosyl + 340.101 Da (K).

### Circular dichroism

Circular dichroism spectra were measured using a J-810 spectrapolarimeter (Jasco, Easton, MD) with a 2 mm path length quartz cuvette. All measurements were performed at 20 °C. Three scans were averaged to generate each spectrum. A blank spectrum of buffer was collected in the same manner and used for background subtraction. R699 recombinant proteins were analyzed in 0.01 M sodium phosphate, 150 mM NaCl (pH 7.4) and 10% glycerol at a concentration of 0.5 mg ml^−1^. Results represent the mean values from triplicate technical repeats in a single experiment. Each protein was analyzed once.

## Supplementary Information

Supplementary Figures.

Supplementary Table S1.

Supplementary Table S2.

Supplementary Table S3.

Supplementary Table S4.

Supplementary Table S5.

Supplementary Table S6.

Supplementary Table S7.

Supplementary Table S8.

Supplementary Table S9.

## Data Availability

Genomic data were downloaded from NCBI’s RefSeq ftp site, which are publicly available. The analyses results are available at RRID Resource ID: SCR_022140 or https://guolab.shinyapps.io/app-mimivirus-publication/*.* Other data will be available from the corresponding author upon reasonable request.
