# Supplementary material for: Comparative genomic and biochemical analyses identify a collagen galactosylhydroxylysyl glucosyltransferase from Acanthamoeba polyphaga mimivirus
Source: Sci Rep. 2022 Oct 7;12:16806. doi: 10.1038/s41598-022-21197-1 (PMC9546862; doi:10.1038/s41598-022-21197-1)
Supplement: Supplementary file 1 — Supplementary Figures. [file 41598_2022_21197_MOESM1_ESM.pdf]

Supplemental Figures

Comparative genomic and biochemical analyses identify a collagen galactosylhydroxylysyl glucosyltransferase from *Acanthamoeba polyphaga mimivirus*

Wenhui Wu[1,2#], Jeong Seon Kim[1#], Aaron O. Bailey[3], William K. Russell[3], Stephen J. Richards[1], Tiantian Chen[1], Tingfei Chen[1], Zhenhang Chen[4], Bo Liang[4], Mitsuo Yamauchi[5], Houfu Guo[1]*

[1]Department of Molecular and Cellular Biochemistry, University of Kentucky, Lexington, KY, USA; Markey Cancer Center, University of Kentucky, Lexington, KY, USA.
[2]Current address: Arvinas, LLC, 5 Science Park, New Haven, CT, USA.
[3]Department of Biochemistry and Molecular Biology, University of Texas Medical Branch, Galveston, TX, USA
[4]Department of Biochemistry, Emory University School of Medicine, Atlanta, GA, USA.
[5]Division of Oral and Craniofacial Health Sciences, Adams School of Dentistry, University of North Carolina at Chapel Hill, Chapel Hill, NC, USA.

#Authors contributed equally

*Correspondence: H.G. (houfu.guo@uky.edu)

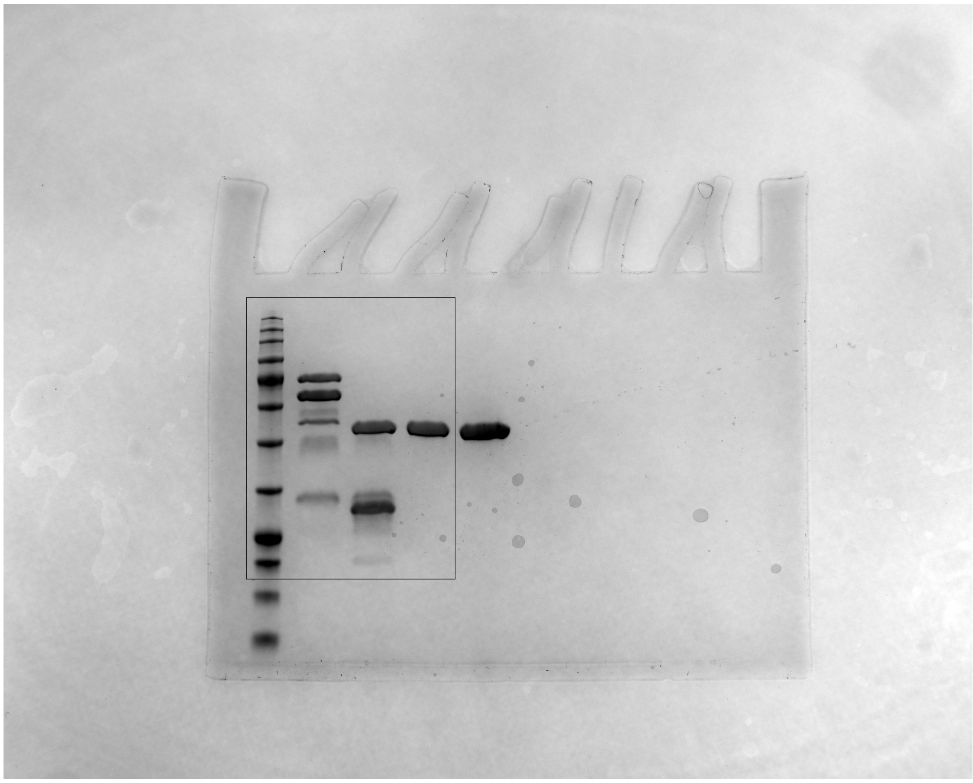

Figure S1. Uncut SDS-polyacrylamide gel image for Fig. 3A. SDS-polyacrylamide gel electrophoresis of R699 protein after IMAC, PreScission cleavage, reverse IMAC. R699 was purified close to homogeneity after 3-step purification. The area shown in Fig. 3A was indicated with a black square.

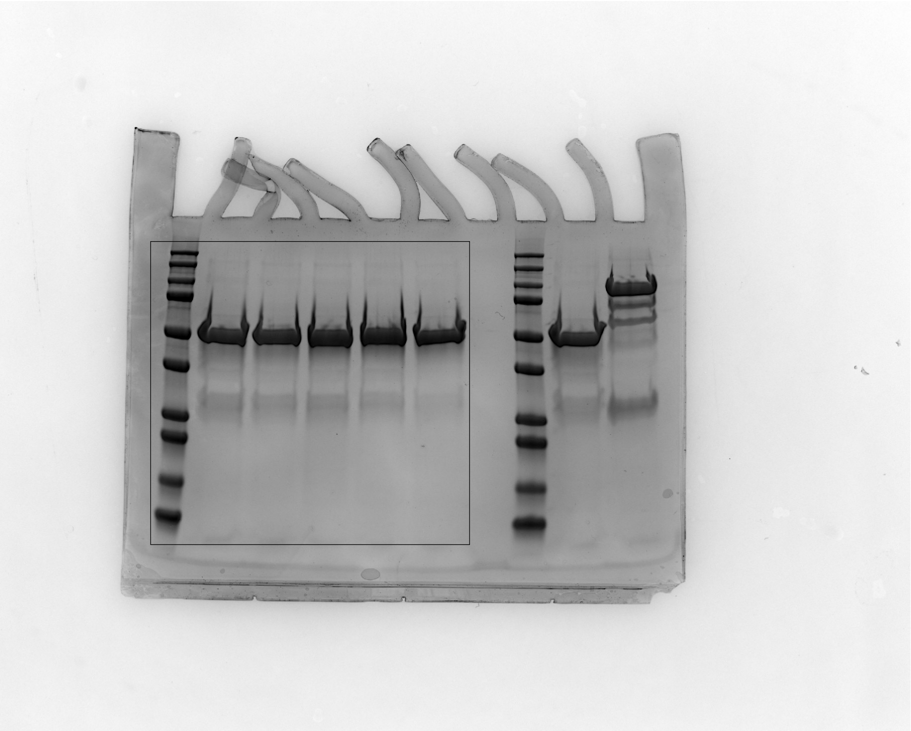

Figure S2. Uncut SDS-polyacrylamide gel image for Fig. 3F. SDS-polyacrylamide gel electrophoresis of R699 wild type (WT) and mutant proteins after IMAC. The area shown in Fig. 3F was indicated with a black square.

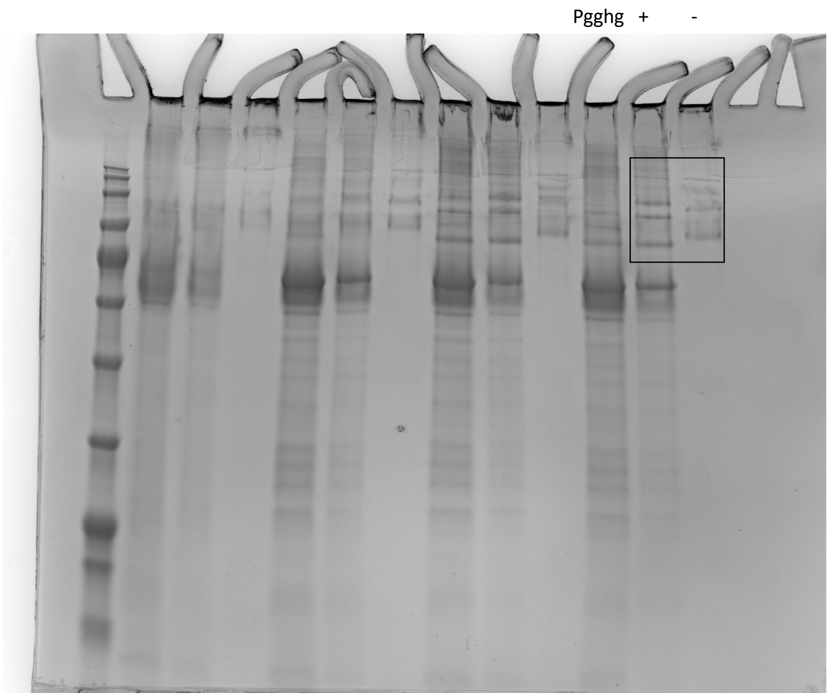

Figure S3. Uncut SDS-polyacrylamide gel image for Fig. 4A. Type IV collagen that had been pre-treated with wild-type (+) protein glucosylgalactosylhydroxylysine glucosidase (PGGHG) or sham-treated (-) was analyzed using SDS-polyacrylamide gel electrophoresis. The area shown in Fig. 4A was indicated with a black square.
