# Supplementary material for: Comparative genomic and biochemical analyses identify a collagen galactosylhydroxylysyl glucosyltransferase from Acanthamoeba polyphaga mimivirus
Source: Sci Rep. 2022 Oct 7;12:16806. doi: 10.1038/s41598-022-21197-1 (PMC9546862; doi:10.1038/s41598-022-21197-1)
Supplement: Supplementary file 6 — Supplementary Table S5. [file 41598_2022_21197_MOESM6_ESM.pdf]

Table_5S: All significant GO BP Terms

id	source	term_id	term_name	term_size	intersection_size	p_value
1	GO:BP	GO:0000209	protein polyubiquitination	327	88	5.1e−27
2	GO:BP	GO:0006457	protein folding	195	49	1.0e−12
3	GO:BP	GO:0051085	chaperone cofactor−dependent protein refolding	24	17	6.1e−12
4	GO:BP	GO:0031146	SCF−dependent proteasomal ubiquitin−dependent protein catabolic process	93	32	8.9e−12
5	GO:BP	GO:0051084	'de novo' posttranslational protein folding	29	17	6.6e−10
6	GO:BP	GO:0006458	'de novo' protein folding	33	18	7.2e−10
7	GO:BP	GO:0061077	chaperone−mediated protein folding	53	22	2.2e−09
8	GO:BP	GO:0070936	protein K48−linked ubiquitination	55	22	5.4e−09
9	GO:BP	GO:0030198	extracellular matrix organization	320	55	2.9e−07
10	GO:BP	GO:0043062	extracellular structure organization	321	55	3.2e−07
11	GO:BP	GO:0048524	positive regulation of viral process	100	26	3.8e−06
12	GO:BP	GO:0006260	DNA replication	227	42	4.7e−06
13	GO:BP	GO:0035966	response to topologically incorrect protein	177	36	4.9e−06
14	GO:BP	GO:0046777	protein autophosphorylation	209	39	1.4e−05
15	GO:BP	GO:0042026	protein refolding	19	11	1.4e−05
16	GO:BP	GO:0048705	skeletal system morphogenesis	88	23	2.9e−05
17	GO:BP	GO:0090084	negative regulation of inclusion body assembly	10	8	4.1e−05
18	GO:BP	GO:0001501	skeletal system development	281	46	4.6e−05
19	GO:BP	GO:0000086	G2/M transition of mitotic cell cycle	238	41	6.2e−05
20	GO:BP	GO:0044839	cell cycle G2/M phase transition	257	43	6.9e−05
21	GO:BP	GO:0048704	embryonic skeletal system morphogenesis	37	14	1.3e−04
22	GO:BP	GO:0009952	anterior/posterior pattern specification	95	23	1.4e−04
23	GO:BP	GO:0006986	response to unfolded protein	155	30	3.4e−04
24	GO:BP	GO:0017157	regulation of exocytosis	147	29	3.6e−04
25	GO:BP	GO:0070979	protein K11−linked ubiquitination	29	12	3.6e−04
26	GO:BP	GO:0006301	postreplication repair	52	16	4.2e−04
27	GO:BP	GO:0090083	regulation of inclusion body assembly	13	8	9.7e−04
28	GO:BP	GO:0046782	regulation of viral transcription	62	17	1.1e−03
29	GO:BP	GO:0006904	vesicle docking involved in exocytosis	32	12	1.3e−03
30	GO:BP	GO:0018105	peptidyl−serine phosphorylation	266	41	1.4e−03
31	GO:BP	GO:0050792	regulation of viral process	210	35	1.5e−03
32	GO:BP	GO:0048706	embryonic skeletal system development	50	15	1.5e−03
33	GO:BP	GO:0043903	regulation of symbiotic process	222	36	2.0e−03
34	GO:BP	GO:0006261	DNA−dependent DNA replication	142	27	2.0e−03
35	GO:BP	GO:0009262	deoxyribonucleotide metabolic process	28	11	2.3e−03
36	GO:BP	GO:0006289	nucleotide−excision repair	105	22	3.9e−03
37	GO:BP	GO:0006354	DNA−templated transcription, elongation	106	22	4.6e−03
38	GO:BP	GO:0034976	response to endoplasmic reticulum stress	269	40	4.9e−03
39	GO:BP	GO:0003002	regionalization	158	28	5.6e−03
40	GO:BP	GO:0018126	protein hydroxylation	25	10	5.9e−03
41	GO:BP	GO:0030433	ubiquitin−dependent ERAD pathway	77	18	6.5e−03
42	GO:BP	GO:0018209	peptidyl−serine modification	284	41	7.9e−03
43	GO:BP	GO:0071897	DNA biosynthetic process	152	27	8.0e−03
44	GO:BP	GO:0009408	response to heat	128	24	1.1e−02
45	GO:BP	GO:0006283	transcription−coupled nucleotide−excision repair	72	17	1.1e−02
46	GO:BP	GO:1904355	positive regulation of telomere capping	17	8	1.4e−02
47	GO:BP	GO:0050434	positive regulation of viral transcription	39	12	1.5e−02
48	GO:BP	GO:0085020	protein K6−linked ubiquitination	9	6	1.6e−02
49	GO:BP	GO:0036503	ERAD pathway	99	20	2.0e−02
50	GO:BP	GO:0048598	embryonic morphogenesis	307	42	2.4e−02
51	GO:BP	GO:0030199	collagen fibril organization	41	12	2.6e−02
52	GO:BP	GO:0035967	cellular response to topologically incorrect protein	155	26	3.5e−02
53	GO:BP	GO:0034605	cellular response to heat	111	21	3.6e−02
54	GO:BP	GO:0070841	inclusion body assembly	19	8	3.9e−02
55	GO:BP	GO:0009266	response to temperature stimulus	156	26	4.0e−02
56	GO:BP	GO:0006368	transcription elongation from RNA polymerase II promoter	79	17	4.0e−02
57	GO:BP	GO:1900034	regulation of cellular response to heat	79	17	4.0e−02
58	GO:BP	GO:1901796	regulation of signal transduction by p53 class mediator	167	27	4.8e−02

g:Profiler (biit.cs.ut.ee/gprofiler)
