# Supplementary material for: Comparative genomic and biochemical analyses identify a collagen galactosylhydroxylysyl glucosyltransferase from Acanthamoeba polyphaga mimivirus
Source: Sci Rep. 2022 Oct 7;12:16806. doi: 10.1038/s41598-022-21197-1 (PMC9546862; doi:10.1038/s41598-022-21197-1)
Supplement: Supplementary file 7 — Supplementary Table S6. [file 41598_2022_21197_MOESM7_ESM.pdf]

Table_6S: All significant GO CC Terms

id	source	term_id	term_name	term_size	intersection_size		p_value
1	GO:CC	GO:0005581	collagen trimer	32	29		4.6e−29
2	GO:CC	GO:0000151	ubiquitin ligase complex	275	79		8.1e−28
3	GO:CC	GO:0098644	complex of collagen trimers	20	19		1.8e−19
4	GO:CC	GO:0031461	cullin−RING ubiquitin ligase complex	161	50		9.6e−19
5	GO:CC	GO:0005788	endoplasmic reticulum lumen	282	64		2.5e−16
6	GO:CC	GO:0019005	SCF ubiquitin ligase complex	60	28		2.4e−15
7	GO:CC	GO:0031463	Cul3−RING ubiquitin ligase complex	36	18		4.2e−10
8	GO:CC	GO:0098643	banded collagen fibril	11	10		4.8e−09
9	GO:CC	GO:0005583	fibrillar collagen trimer	11	10		4.8e−09
10	GO:CC	GO:0098651	basement membrane collagen trimer	9	9		7.1e−09
11	GO:CC	GO:0098645	collagen network	8	8		1.1e−07
12	GO:CC	GO:0098642	network−forming collagen trimer	8	8		1.1e−07
13	GO:CC	GO:0005587	collagen type IV trimer	7	7		1.7e−06
14	GO:CC	GO:0005604	basement membrane	59	17		4.4e−05
15	GO:CC	GO:0005663	DNA replication factor C complex	5	5		3.9e−04
16	GO:CC	GO:0008540	proteasome regulatory particle, base subcomplex	12	7		9.9e−04
17	GO:CC	GO:0032593	insulin−responsive compartment	9	6		1.8e−03
18	GO:CC	GO:0030139	endocytic vesicle	254	36		3.3e−03
19	GO:CC	GO:0005657	replication fork	62	14		1.2e−02
20	GO:CC	GO:0055037	recycling endosome	140	23		1.3e−02
21	GO:CC	GO:0045335	phagocytic vesicle	98	18		2.0e−02
22	GO:CC	GO:0034663	endoplasmic reticulum chaperone complex	5	4		2.8e−02
23	GO:CC	GO:0031371	ubiquitin conjugating enzyme complex	9	5		4.0e−02
24	GO:CC	GO:0016607	nuclear speck	320	39		4.5e−02
25	GO:CC	GO:0005665	RNA polymerase II, core complex	14	6		4.9e−02

g:Profiler (biit.cs.ut.ee/gprofiler)
