# Supplementary material for: Comparative genomic and biochemical analyses identify a collagen galactosylhydroxylysyl glucosyltransferase from Acanthamoeba polyphaga mimivirus
Source: Sci Rep. 2022 Oct 7;12:16806. doi: 10.1038/s41598-022-21197-1 (PMC9546862; doi:10.1038/s41598-022-21197-1)
Supplement: Supplementary file 8 — Supplementary Table S7. [file 41598_2022_21197_MOESM8_ESM.pdf]

Table_7S: All significant GO MF Terms

id	source	term_id	term_name	term_size	intersection_size		p_value
1	GO:MF	GO:0030020	extracellular matrix structural constituent conferring tensile strength	41	39		3.2e−41
2	GO:MF	GO:0005201	extracellular matrix structural constituent	167	66		1.1e−31
3	GO:MF	GO:0016887	ATPase activity	316	85		6.1e−27
4	GO:MF	GO:0061650	ubiquitin−like protein conjugating enzyme activity	42	32		7.0e−27
5	GO:MF	GO:0061631	ubiquitin conjugating enzyme activity	40	31		2.1e−26
6	GO:MF	GO:0004386	helicase activity	101	39		1.1e−17
7	GO:MF	GO:0019003	GDP binding	66	30		1.1e−15
8	GO:MF	GO:0004707	MAP kinase activity	20	17		4.8e−15
9	GO:MF	GO:0005524	ATP binding	285	63		8.1e−15
10	GO:MF	GO:0004693	cyclin−dependent protein serine/threonine kinase activity	23	18		8.2e−15
11	GO:MF	GO:0097472	cyclin−dependent protein kinase activity	23	18		8.2e−15
12	GO:MF	GO:0030554	adenyl nucleotide binding	340	64		1.6e−11
13	GO:MF	GO:0032559	adenyl ribonucleotide binding	332	63		1.8e−11
14	GO:MF	GO:0031489	myosin V binding	15	13		2.5e−11
15	GO:MF	GO:0008094	DNA−dependent ATPase activity	88	29		1.1e−10
16	GO:MF	GO:0140097	catalytic activity, acting on DNA	178	41		9.1e−10
17	GO:MF	GO:0003924	GTPase activity	250	49		3.8e−09
18	GO:MF	GO:0032550	purine ribonucleoside binding	204	42		2.4e−08
19	GO:MF	GO:0032549	ribonucleoside binding	204	42		2.4e−08
20	GO:MF	GO:0001883	purine nucleoside binding	205	42		2.8e−08
21	GO:MF	GO:0003724	RNA helicase activity	43	18		4.1e−08
22	GO:MF	GO:0003678	DNA helicase activity	59	21		4.3e−08
23	GO:MF	GO:0001882	nucleoside binding	208	42		4.5e−08
24	GO:MF	GO:0019001	guanyl nucleotide binding	217	43		5.1e−08
25	GO:MF	GO:0032561	guanyl ribonucleotide binding	216	42		1.6e−07
26	GO:MF	GO:0016706	2−oxoglutarate−dependent dioxygenase activity	48	18		3.6e−07
27	GO:MF	GO:0031072	heat shock protein binding	95	24		5.3e−06
28	GO:MF	GO:0001671	ATPase activator activity	20	11		5.8e−06
29	GO:MF	GO:0050321	tau−protein kinase activity	20	11		5.8e−06
30	GO:MF	GO:0140098	catalytic activity, acting on RNA	330	50		2.3e−05
31	GO:MF	GO:0004683	calmodulin−dependent protein kinase activity	19	10		4.8e−05
32	GO:MF	GO:0000287	magnesium ion binding	146	29		5.4e−05
33	GO:MF	GO:0060590	ATPase regulator activity	34	13		6.5e−05
34	GO:MF	GO:0051787	misfolded protein binding	24	11		6.7e−05
35	GO:MF	GO:0051082	unfolded protein binding	93	22		7.7e−05
36	GO:MF	GO:0051213	dioxygenase activity	67	18		1.3e−04
37	GO:MF	GO:0005525	GTP binding	191	33		2.3e−04
38	GO:MF	GO:0015036	disulfide oxidoreductase activity	32	12		2.6e−04
39	GO:MF	GO:0016667	oxidoreductase activity, acting on a sulfur group of donors	50	15		2.8e−04
40	GO:MF	GO:0051087	chaperone binding	86	20		3.8e−04
41	GO:MF	GO:0030332	cyclin binding	29	11		6.8e−04
42	GO:MF	GO:0048407	platelet−derived growth factor binding	11	7		8.1e−04
43	GO:MF	GO:0000405	bubble DNA binding	8	6		1.2e−03
44	GO:MF	GO:0044183	protein folding chaperone	20	9		1.2e−03
45	GO:MF	GO:0001228	DNA−binding transcription activator activity, RNA polymerase II−specific	344	47		1.2e−03
46	GO:MF	GO:0030544	Hsp70 protein binding	31	11		1.5e−03
47	GO:MF	GO:0001216	DNA−binding transcription activator activity	347	47		1.6e−03
48	GO:MF	GO:0044389	ubiquitin−like protein ligase binding	281	40		2.5e−03
49	GO:MF	GO:0016705	oxidoreductase activity, acting on paired donors, with incorporation or reduction of molecular oxygen	148	26		2.7e−03
50	GO:MF	GO:0031625	ubiquitin protein ligase binding	264	38		3.3e−03
51	GO:MF	GO:0003899	DNA−directed 5'−3' RNA polymerase activity	28	10		3.9e−03
52	GO:MF	GO:0009378	four−way junction helicase activity	6	5		4.0e−03
53	GO:MF	GO:0008353	RNA polymerase II CTD heptapeptide repeat kinase activity	18	8		4.9e−03
54	GO:MF	GO:0061630	ubiquitin protein ligase activity	257	36		1.1e−02
55	GO:MF	GO:0017022	myosin binding	58	14		1.1e−02
56	GO:MF	GO:0097747	RNA polymerase activity	31	10		1.1e−02
57	GO:MF	GO:0034062	5'−3' RNA polymerase activity	31	10		1.1e−02
58	GO:MF	GO:0043138	3'−5' DNA helicase activity	16	7		2.1e−02
59	GO:MF	GO:0008301	DNA binding, bending	16	7		2.1e−02
60	GO:MF	GO:0048156	tau protein binding	40	11		2.3e−02
61	GO:MF	GO:0061659	ubiquitin−like protein ligase activity	267	36		2.4e−02
62	GO:MF	GO:0008474	palmitoyl−(protein) hydrolase activity	12	6		3.0e−02
63	GO:MF	GO:0015037	peptide disulfide oxidoreductase activity	12	6		3.0e−02
64	GO:MF	GO:0015035	protein disulfide oxidoreductase activity	12	6		3.0e−02
65	GO:MF	GO:0017116	single−stranded DNA helicase activity	17	7		3.3e−02
66	GO:MF	GO:0002039	p53 binding	57	13		4.1e−02
67	GO:MF	GO:0008559	ATPase−coupled xenobiotic transmembrane transporter activity	5	4		5.0e−02

g:Profiler (biit.cs.ut.ee/gprofiler)
